# Posteromedial corner injuries result in the same posterior translation as posterolateral corner injuries in PCL ruptures

**DOI:** 10.1002/jeo2.70118

**Published:** 2024-12-18

**Authors:** Olivia Bohe, Frederik Greve, Svenja Höger, Julian Mehl, Sebastian Siebenlist, Lukas Willinger

**Affiliations:** ^1^ Department of Sports Orthopedics Technical University of Munich Munich Germany; ^2^ Department of Trauma Surgery Technical University of Munich Munich Germany

**Keywords:** posterior cruciate ligament, posterior tibial translation, posterolateral corner, posteromedial corner

## Abstract

**Purpose:**

Ruptures of the posterior cruciate ligament (PCL) are often accompanied by posterolateral corner (PLC) and posteromedial corner (PMC) injuries. This study investigates the incidence and impact of PMC and PLC injuries on posterior tibial translation (PTT). It was hypothesized that PMC injuries are more common and impactful than previously reported.

**Methods:**

In this retrospective study, all patients with a PCL injury between January 2016 and December 2023 and received magnetic resonance imaging (MRI) within 30 days of trauma were included. Patients with atraumatic PCL instability, missing MRI or additional anterior cruciate ligament (ACL) rupture were excluded. Posttraumatic MRI was analyzed for peripheral injuries. Preoperative stress radiographs for PTT were measured, and the side‐to‐side difference was calculated. The statistical significance level was set at *p* < 0.05.

**Results:**

Ninety‐two patients were included, predominantly male (71.7%) with a mean age of 35.8 ± 15.6 years at injury. The mean time from injury to MRI was 7.3 ± 7.9 days. There were 16 patients (27.4%) with isolated PCL injuries. The prevalence of medial injuries (37.0% superficial medial collateral ligament [sMCL], 66.3% deep medial collateral ligament and 51.1% posterior oblique ligament [POL]) was comparable to lateral injuries (22.8% LCL, 55.4% PLC and 13.0% popliteus tendon). Injuries of the PMC (sMCL ± POL) occurred in 53 patients (57.6%) and of the PLC (POP ± PLC ± LCL) in 59 (64.1%) patients. PTT was significantly increased in the presence of a peripheral‐associated injury compared to isolated PCL injury (*p* < 0.01). With a combined injury of PMC + PLC the PTT was significantly larger than in the case of a unilateral injury (*p* < 0.05 compared to PLC; *p* < 0.05 compared to PMC).

**Conclusion:**

PCL injuries are commonly associated with PMC and/or PLC injuries. A PTT of >10 mm is equally caused by PLC and PMC‐associated injuries. Knowledge about the severity and localization of peripheral‐associated injuries is therefore essential for therapeutic decision‐making.

**Level of Evidence:**

Level III retrospective cohort study.

**Abbreviations**
ACL: anterior cruciate ligament
dMCL: deep medial collateral ligament
LCL: lateral collateral ligament
MRI: magnetic resonance imaging
n.s.: not significant
PCL: posterior cruciate ligament
PLC: posterolateral corner
PMC: posteromedial corner
POL: posterior oblique ligament
POP: popliteus tendon
PTT: posterior tibial translation
SD: standard deviation
sMCL: superficial medial collateral ligament
SSD: side‐to‐side difference

## BACKGROUND

Posterior cruciate ligament (PCL) ruptures are common knee joint injuries, resulting from high‐energy impacts such as car accidents, sports‐related injuries or low‐velocity traumas [9]. The PCL, together with the anterior cruciate ligament (ACL), is essential for knee stability, particularly in preventing the tibia from moving backwards relative to the femur and controlling varus rotation [2, 8]. While PCL injuries occur less frequently than ACL tears, they can cause significant functional impairment and long‐term knee instability if not properly managed [9].

Research has well established that PCL injuries often coincide with injuries to the posterolateral corner (PLC), which includes the lateral collateral ligament (LCL), popliteus tendon (POP), popliteofibular ligament and posterolateral capsule [16, 30]. Recently, there has been increasing attention on associated injuries to the posteromedial corner (PMC), specifically involving the medial collateral ligament (MCL) and the posterior oblique ligament (POL) [18, 22, 24].

Despite their clinical significance, PMC injuries are not recognized in the most commonly used classification systems, namely the Harner classification for acute PCL injuries or the Fanelli classification for acute PCL injuries which only acknowledges the relevance of PLC injuries [8, 12].

However, both biomechanical and clinical studies have demonstrated that PMC structures play a crucial role in the rotational stability of the knee. Cadaveric biomechanical studies have shown that the PMC and especially the posteromedial capsule with the POL are significant stabilizers for posterior translation and tibial internal rotation in the knee, mainly in full knee extension [24, 27, 34].

The goal of this study was to examine the incidence and characteristics of concomitant peripheral injuries in combination with PCL ruptures, focusing on both posterolateral (PL) and posteromedial (PM) structures. Second, we aimed to assess the significance of concomitant PM and PL injuries in contributing to increased posterior tibial translation (PTT).

It was hypothesized that PMC injuries occur more frequently than previously reported in the literature and that their impact on PTT is as relevant as that of PLC injuries and result in an PTT > 10 mm side‐to‐side difference (SSD) in lateral stress radiographs.

## MATERIALS AND METHODS

### Data collection

**Figure 1:**
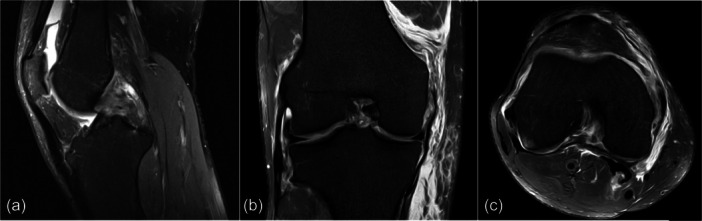
Exemplary representation of T2‐weighted MRI images depicting: (a) typical case of an acutely ruptured posterior cruciate ligament on sagittal view, (b) III femoral rupture of superficial medial collateral ligament on coronal view and (c) rupture of posterior oblique ligament on axial view. MRI, magnetic resonance imaging.

The approval by the ethics committee was obtained (study no. 2022‐510‐S‐NP). The study complied with the Declaration of Helsinki and its respective amendments. In this retrospective study, all patients with available preoperative magnetic resonance imaging (MRI) scans who were treated either non‐operatively or surgically for a traumatic PCL injury between January 2016 and December 2023 in a single institution were included (Figure 1a–c). Patients younger than 16 years, patients with additional ACL ruptures, and those for whom the time from injury to MRI scan exceeded 30 days were excluded.

Demographic patient data, such as age, sex, date of injury, date of MRI, date and type of treatment, and data about the trauma mechanism were collected from the medical electronic reporting system.

Posttraumatic MRI was thoroughly analyzed for associated peripheral injuries by two sports medicine‐trained orthopaedic surgeons. The images were acquired from multiple centres as patients typically bring their MRIs from outpatient institutes. Images were analyzed if they met the minimum criteria of (1) field strength higher than 1.5 T, (2) STIR or T2‐weighted images in all three planes and (3) slice thickness of 3 mm or less.

Injuries to the superficial medial collateral ligament (sMCL) were localized (tibial, femoral and intraligamentous ruptures) and graded according to the criteria suggested by Rasenberg et al. [25]. Mild (Grade I) injuries were defined by high signal intensity or oedema in the T2‐weighted images without disruption of the ligament; moderate (Grade II) injuries were characterized by partial discontinuity with increased signal intensity within the ligament on T2‐weighted images and in severe (Grade III) injuries the sMCL shows a complete disruption often with a high signal intensity gap visible on T2‐weighted images, often accompanied by oedema or surrounding haemorrhage [25].

The deep medial collateral ligament (dMCL) was considered as ruptured, when there was complete discontinuity in the dMCL fibres and there was fluid between the dMCL and sMCL. The location of the dMCL rupture was classified as meniscofemoral or meniscotibial. Furthermore, the POL or the posterolateral capsule were considered injured when there was any oedema around the respective structures present in T2 weighted imaging.

The LCL with the location and grade of ruptures (fibular, femoral or intraligamentous rupture) was graded equally to sMCL injuries. Injuries of the POP were defined as the presence of oedema or a partial or complete discontinuity at the femoral attachment.

For the purpose of analysis, PMC injuries were defined as injuries to the sMCL, and/or the POL. PLC injuries were defined as lesions of POP, LCL and/or posterolateral capsule structures.

Additionally, for those available and for those not acutely operated on, preoperative stress radiographs for PTT at 90° knee flexion with 15 kP (Telos‐Stress‐Device) were evaluated for PTT on lateral radiographs as previously described and the SSD was calculated [14]. Stress radiographs are advocated to diagnose and grade the severity of PCL injuries as they provide an objective assessment of PTT in comparison to the healthy, contralateral knee [14, 15].

The posterior tibial slope (PTS) was also measured using lateral knee stress radiographs because it was described to influence PTT [10]. The tibial proximal anatomical axis (TPAA) was determined by drawing a line connecting the midpoints of two outer diameter circles in the proximal tibial shaft. Subsequently, the angle between the TPAA and the medial tibial plateau was measured [36].

### Statistical analysis

Data was analyzed using the Statistical Package for the Social Sciences (SPSS, version 27; IBM Inc.). Patients' characteristics were described using mean and standard deviation (SD) for continuous variables. Absolute numbers and relative percentages were used for categorical variables. The normality of data was tested prior to further assessment using the Kolmogorov–Smirnoff test. Significances were calculated using paired *t* tests or Mann–Whitney *U* test when appropriate. Comparisons between means of PTT and PTS were conducted using one‐way analysis of variance with post hoc analysis with Tukey's honestly significant difference test to control for Type I error across multiple pairwise comparisons. A post hoc power analysis was performed to assess the differences in PTT and PTS between the groups, with a significance level set at 0.05. The calculated power was 81.1% (G*Power Version 3.1.9.6). *p* Values < 0.05 were considered statistically significant.

Cohen's Kappa was calculated for the two raters' categorical assessments of MRI. Interobserver reliability was good to excellent with *κ* = 0.77–0.97, indicating substantial agreement between raters.

## RESULTS

### Demographic data

Ninety‐two patients (71.7% [*n* = 66] male) were included. The mean age at injury was 35.8 ± 15.6 years, and the mean BMI was 25.9 ± 4.7 kg/m^2^.

The mechanism of injury was sports‐related in 33.7% (*n* = 31) of patients, traffic accidents (e.g., bicycling and car) in 41.3% (*n* = 38), knee distortion or fall from standing height in 18 (19.6%) patients and other high‐impact trauma (e.g., fall from heights over 2 m) in 5 (5.4%) patients.

Surgery was performed in 70 patients (76.1%). Of these, 12 patients (17.1%) received isolated PCL reconstruction, 21 patients (30.0%) underwent PCL reconstruction with posterolateral reconstruction using the Arciero technique, while 17 patients (24.3%) had PCL reconstruction with acute MCL refixation. Twenty patients (28.6%) underwent fixation of a tibial bony PCL avulsion injury.

### MRI findings

The mean time from injury to MRI was 7.3 ± 7.9 days. Bony avulsions of the PCL were present in 28 (30.4%) patients.

Eighteen patients (19.6%) had isolated PCL ruptures. On the medial side of the knee, 34 patients (37.0%) exhibited sMCL ruptures, thereof 23 (25.0%) femoral avulsions, 6 (6,5%) tibial avulsions and 5 (5.4%) intraligamentous ruptures. There were 7 (7.6%) Grade I ruptures, 10 (10.9%) Grade II ruptures and 17 (18.5%) Grade III ruptures. Sixty‐one patients (66.3%) exhibited dMCL ruptures, and 47 patients (51.1%) exhibited injuries to the POL. Overall, the PMC was injured in 53 (57.6%) patients, including 6 (6.5%) isolated sMCL injuries and 19 (20.7%) isolated POL injuries. Combined injuries to the sMCL and POL were present in 28 (30.4%) patients.

On the lateral side, there were 21 (22.8%) injuries of the LCL, with 8 (8.7%) femoral‐sided, 5 (5.4%) tibial‐sided and 8 (8.7%) intraligamentous injuries. There were 9 (9.8%) Grade I injuries, 8 (8.7%) Grade II and 4 (4.3%) Grade III injuries.

The POP was injured in 12 (13.0%) patients, and 51 patients (55.4%) had injuries of the posterolateral capsule complex.

Consequently, the PLC was injured in 59 patients (64.1%): isolated injuries of the LCL were present in 5 (5.4%) patients, isolated POP injuries in 2 (2.2%) patients and isolated posterolateral capsule injuries were present in 33 (35.9%) patients. Combined injuries with all three structures (LCL, POP and posterolateral capsule complex) were present in six (6.5%) patients. One (1.1%) patient had injuries of the POP and the LCL, three (3.3%) patients had injuries of the POP and the posterolateral capsule complex and nine patients (9.8%) of the LCL and posterolateral capsule complex (Table 1).

**Table 1:** List detailing the frequency of injuries to the peripheral anatomical structures around the knee.

	Total number (in %)
PMC	
PMC injuries overall	53 (57.6)
Isolated sMCL	6 (6.5)
Isolated POL	19 (20.7)
Combined PMC (POL + sMCL)	28 (30.4)
PLC	
PLC injuries overall	59 (64.1)
Isolated LCL	5 (5.4)
Isolated POP	2 (2.2)
Isolated posterolateral capsule	33 (35.9)
Combined LCL + POP	1 (1.1)
Combined LCL + posterolateral capsule	9 (9.8)
Combined POP + posterolateral capsule	3 (3.3)
Complete PLC (POP + LCL + posterolateral capsule)	6 (6.5)
Overall	
Isolated PCL	18 (19.6)
Isolated PMC	20 (21.7)
Isolated PLC	21 (22.8)
Combined PMC + PLC	33 (35.9)

Abbreviations: LCL, lateral collateral ligament; PCL, posterior cruciate ligament; PLC, posterolateral corner; PMC, posteromedial corner; POL, posterior oblique ligament; POP, popliteus tendon; sMCL, superficial medial collateral ligament.

There was no significant difference in the frequency of PMC or PLC injuries (n.s.).

### Posterior tibial translation and posterior tibial slope

Forty‐one patients (44.7%) received lateral stress radiographs for PTT, of whom 19 patients received radiographs after 3 months of conservative management and 22 patients presented delayed after initial management in a different clinic. The mean time from injury to stress radiographs was 211.7 ± 466.4 days.

The mean PTT on the injured side was 12.7 ± 3.5 mm compared to 2.6 ± 1.9 mm on the uninjured side. This resulted in a mean SSD in PTT of 9.8 ± 3.3 mm overall.

Patients with isolated PCL injuries had a mean SSD in PTT of 5.7 ± 2.6 mm, patients with medial‐sided ligament injuries of 9.6 ± 2.0 mm (compared to isolated PCL injury; *p* < 0.05) and patients with lateral and posterolateral injuries of 9.5 ± 2.4 mm (compared to isolated PCL injury; *p* < 0.05). Of the 18 patients who had an SSD in PTT of ≥10 mm, there were no isolated PCL injuries. Two of those patients (11.1%) had concomitant PMC injuries, 3 (16.7%) had concomitant PLC injuries, and 13 (72.2%) patients had combined PMC/PLC injuries. When patients had both PMC and PLC injuries (SSD: 11.8 ± 3.0 mm) the posterior translation was significantly higher compared to patients with isolated PCL injuries (*p* < 0.01). Additionally, combined PMC and PLC injuries exhibited a significantly larger PTT compared to isolated PMC (*p* < 0.05) and PLC injuries (*p* < 0.05).

**Figure 2:**
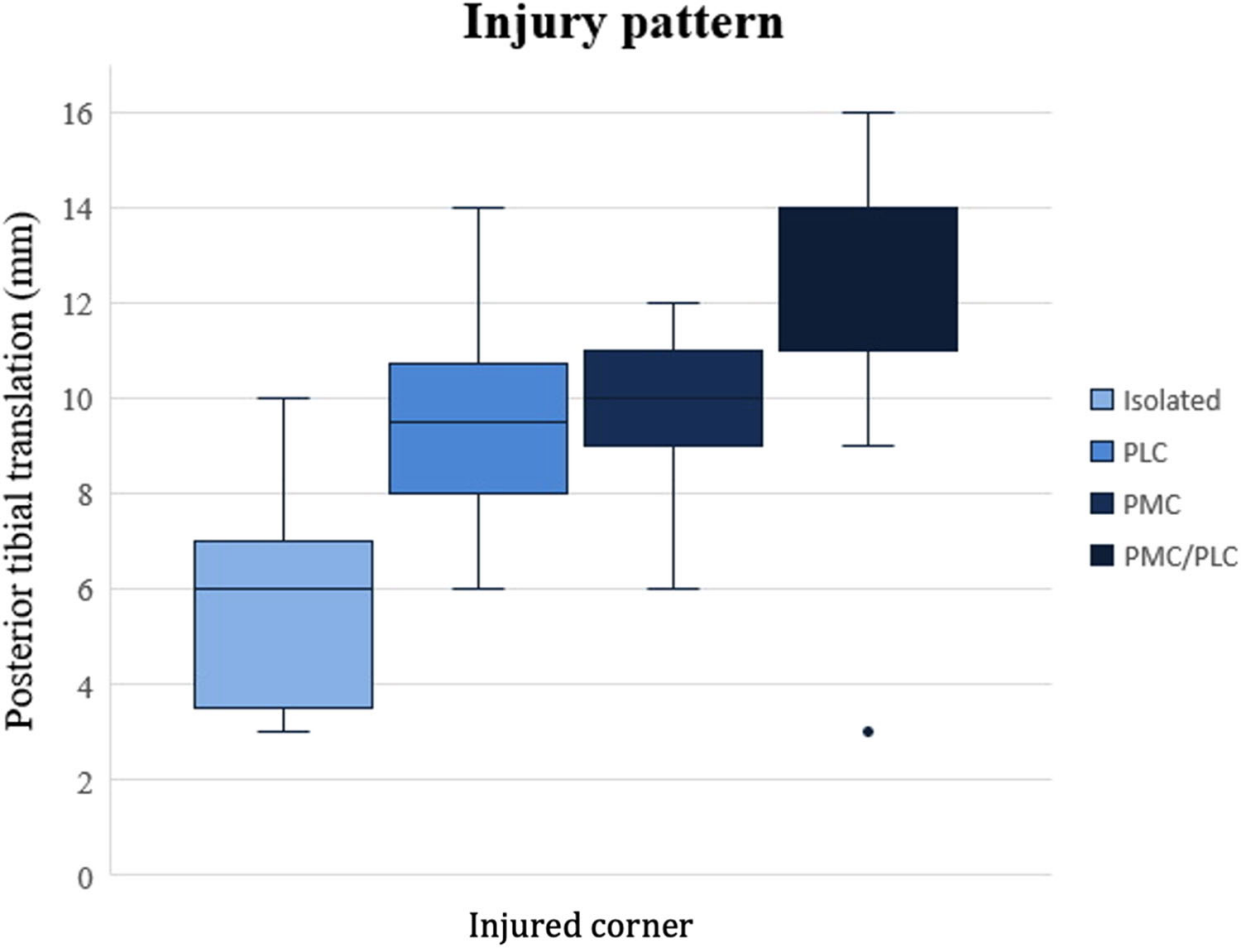
Box plot displaying the mean posterior tibial translation across groups with isolated PCL injuries, either concomitant PLC or PMC injuries, and injuries involving both PMC and PLC. PCL, posterior cruciate ligament; PLC, posterolateral corner; PMC, posteromedial corner.

However, when comparing mean PTT between patients with PMC versus PLC injuries there was no significant difference (n.s., Figure 2).

Overall, the mean PTS was 7.5 ± 1.7° (range: 3–10°). Patients with isolated PCL injuries exhibited a PTS of 7.6 ± 1.1°. In patients with PMC injuries, the PTS was 7.0 ± 2.2°, with PLC injuries 7.9 ± 1.4° and in combined PMC/PLC injuries, the mean PTS was 7.4 ± 1.8° (all n.s.). There was no significant correlation between PTS and PTT (n.s.).

## DISCUSSION

The main findings of this study indicate that concomitant PMC and/or PLC injuries occur in the majority of patients with PCL ruptures with nearly the same frequency. Importantly, PMC and PLC injuries result in almost identically increased PTT, which was significantly higher compared to isolated PCL ruptures. An injury to both peripheral sides had an even more pronounced effect on PTT. This study shows that injuries to both the PMC and PLC structures could lead to a significant PTT and must be diagnosed and addressed in the treatment of PCL injuries.

There is a growing interest in the study of concomitant peripheral injuries accompanying cruciate ligament tears. Over the last years, the vast majority of the literature has focused on peripheral injuries in ACL tears, especially the anterolateral and anteromedial complex. It has been shown that isolated ACL injuries are very rare and that additional peripheral lesions cause increased rotatory instability and lead to a higher rate of ACL failure [1, 28]. With regard to the PCL, many studies have focused on the prevalence and clinical relevance of PLC injuries but there is a notable lack of research on concomitant medial‐sided injuries [3]. It is also well known that PCL injuries rarely occur in isolation, with the incidence of concomitant PLC injuries reported to be approximately 60% [3, 7, 16]. Only very few studies report on the incidence of PMC injuries and estimated the prevalence between 37% and 40% [3, 22]. In the present study, 57.6% of patients demonstrated an injury of the PMC (sMCL and/or POL), which showed an even higher prevalence than previously reported. The incidence might be higher as injured structures were graded and both low‐grade and high‐grade injuries were included in our study. However, the number is similar to concomitant medial‐sided injuries in ACL tears [31, 33].

The impact of peripheral injuries on translational and rotational instability has been described biomechanically. The PMC, including the POL, is a secondary restraint of PTT and tibial internal rotation with a higher contribution near extension [13, 27]. This is due to their length‐change patterns, with the PMC being tight in extension and lax beyond 30° of flexion [34]. Petersen et al. showed that cutting the POL along with sMCL led to a significantly higher PTT in PCL‐injured knees over the entire range of motion [23]. The highest increase in PTT occurred after cutting the POL and PMC. Ritchie et al. attested the largest effect in stabilizing the PCL deficient knee against PTT to the sMCL [26]. Even though this seems controversial, it shows that both structures have a role in controlling PTT and tibial internal rotation. Hence, it is not surprising that a combination of sMCL and POL reconstruction along with PCL reconstruction could restore rotational stability and PTT in combined injured knees [32].

In our study, concomitant PMC injuries significantly increased PTT compared to isolated PCL injuries in lateral stress radiographs in 90° knee flexion.

Interestingly, the PTT was not different between patients with concomitant PLC or PMC injuries, suggesting that the stabilizing function of the PMC is equally as important as the PLC. This is in accordance with biomechanical studies, which showed that the posteromedial structures serve as secondary stabilizers to the knee especially when the PCL is injured [27, 29]. Therefore, overlooked or untreated medial ligament injuries in PCL ruptures can potentially lead to persistent instability and a higher graft failure rate [1, 24, 28].

Furthermore, untreated PMC injuries serve as an independent risk factor for graft failure and increased laxity of the graft after PCL reconstruction [11, 22, 24]. Pizza et al. discovered a significantly higher graft failure rate of PCL‐based multi‐ligament knee injuries, necessitating revision surgery, in patients with additional PMC injuries and reconstruction [24]. Lundquist et al. added that not reconstructing the PMC in PCL reconstruction surgery can lead to increased and accelerated medial osteoarthritis and early failure of PCL reconstruction [18]. Recognizing the significance of PMC injuries is vital for accurate diagnosis and treatment planning in patients with PCL ruptures.

With regard to the PTS, it has been shown that a low PTS is a risk factor for primary PCL ruptures [5, 17, 19]. Interestingly, in the present study no significant differences in PTS between patients with or without peripheral injuries and no correlation between PTS and PTT were observed. Compared to recent studies the mean PTS in our study cohort (7.5 ± 1.7°) was comparable to former studies, where the cut‐off value for a high‐risk was set at approximately 3.9–5.6° [19, 35, 37]. While there are several different measuring techniques for PTS, it is widely agreed that full‐length tibia radiographs are more appropriate to receive reliable values for PTS. Utilizing radiographs with only the proximal part of the tibia depicted, such as in standard true lateral tibia radiographs, can lead to differences in measurement results [6, 20]. However, many previous studies used normal lateral knee radiographs or even dedicated knee MRI scans to measure PTS, and our results are therefore comparable with former studies [4, 5, 10, 19].

This study has several limitations. This is a retrospective study and MRI was conducted on different scanners as many patients bring their scans from external radiology institutions, which could possibly lead to a difference in the quality of the scans. However, the minimum standard of quality defined for inclusion was high, reducing heterogeneous quality. Further, peripheral injuries, especially injuries of the knee joint capsule, are best diagnosed in MRIs taken within a short time span after injury. It has been found that peripheral injuries can hardly be diagnosed when the timespan from injury to MRI exceeds 12 weeks [21]. Therefore, the mean time from injury to MRI scan in our study was only 7.25 days and all patients who received their first MRI 30 days or later after injury were excluded. Lateral stress radiographs were performed in patients after early initial conservative management with PTS‐brace or when patients had a delayed diagnosis without brace management, which could influence PTT as well. MRI scans were analyzed by two independent researchers. In case of disagreement, results of the more senior sports orthopaedic‐trained surgeon with over 10 years' experience were used. However, this could lead to bias in reporting results.

In clinical practice, it is important to distinguish between isolated PCL injuries and, in particular, between PLC and PMC peripheral lesions. Concomitant injuries to the PMC and/or PLC resulted in a significant increase in PTT, and surgeons need to be aware of additional peripheral injuries when the PTT is >9 mm. A comprehensive clinical examination and a detailed review of acutely performed MRIs are important to diagnose concomitant injuries to medial and lateral knee structures.

## CONCLUSION

The study highlights the significant impact of PMC injuries on PTT in patients with PCL ruptures. This finding underscores the importance of recognizing and addressing not only PLC injuries but also PMC injuries in clinical practice. In the case of extensive PTT, a detailed clinical examination is recommended to differentiate between concomitant posterolateral or posteromedial peripheral injuries. Injured structures need to be incorporated into the overall treatment algorithm.

## AUTHOR CONTRIBUTIONS

**Olivia Bohe**: Collection of data, statistical analysis of data and writing of manuscript. **Frederik Greve**: Collection of data and correcting of the manuscript. **Svenja Höger**: Correcting of the manuscript and statistical analysis. **Julian Mehl**: Conception of study and correction of manuscript. **Sebastian Siebenlist**: Conception of study, supervision and correction of manuscript. **Lukas Willinger**: Conception of study, supervision of data collection, interpretation of data and writing of manuscript. All authors read and approved the submitted version of the manuscript.

## CONFLICT OF INTEREST STATEMENT

The authors declare no conflicts of interest.

## ETHICS STATEMENT

The ethics committee of the Technical University Munich approved the study (study no. 2022‐510‐S‐NP). The study complied with the Declaration of Helsinki and its respective amendments.

## Data Availability

The data sets used and/or analyzed during the current study are available from the corresponding author upon reasonable request.

## References

[1] Alm, L. , Drenck, T.C. , Frings, J. , Krause, M. , Korthaus, A. , Krukenberg, A. et al. (2021) Lower failure rates and improved patient outcome due to reconstruction of the MCL and revision ACL reconstruction in chronic medial knee instability. Orthopaedic Journal of Sports Medicine, 9, 2325967121989312. Available from: 10.1177/2325967121989312 33796589 PMC7968026

[2] Amis, A.A. , Bull, A.M.J. , Gupte, C.M. , Hijazi, I. , Race, A. & Robinson, J.R. (2003) Biomechanics of the PCL and related structures: posterolateral, posteromedial and meniscofemoral ligaments. Knee Surgery, Sports Traumatology, Arthroscopy, 11, 271–281. Available from: 10.1007/s00167-003-0410-7 12961064

[3] Anderson, M.A. , Simeone, F.J. , Palmer, W.E. & Chang, C.Y. (2018) Acute posterior cruciate ligament injuries: effect of location, severity, and associated injuries on surgical management. Skeletal Radiology, 47, 1523–1532. Available from: 10.1007/s00256-018-2977-6 29858916

[4] Bernhardson, A.S. , DePhillipo, N.N. , Aman, Z.S. , Kennedy, M.I. , Dornan, G.J. & LaPrade, R.F. (2019) Decreased posterior tibial slope does not affect postoperative posterior knee laxity after double‐bundle posterior cruciate ligament reconstruction. The American Journal of Sports Medicine, 47, 318–323. Available from: 10.1177/0363546518819786 30657698

[5] Bernhardson, A.S. , DePhillipo, N.N. , Daney, B.T. , Kennedy, M.I. , Aman, Z.S. & LaPrade, R.F. (2019) Posterior tibial slope and risk of posterior cruciate ligament injury. The American Journal of Sports Medicine, 47, 312–317. Available from: 10.1177/0363546518819176 30640507

[6] Dean, R.S. , DePhillipo, N.N. , Chahla, J. , Larson, C.M. & LaPrade, R.F. (2021) Posterior tibial slope measurements using the anatomic axis are significantly increased compared with those that use the mechanical axis. Arthroscopy: The Journal of Arthroscopic & Related Surgery, 37, 243–249. Available from: 10.1016/j.arthro.2020.09.006 32949632

[7] Fanelli, G.C. & Edson, C.J. (1995) Posterior cruciate ligament injuries in trauma patients: part II. Arthroscopy: The Journal of Arthroscopic & Related Surgery, 11, 526–529. Available from: 10.1016/0749-8063(95)90127-2 8534292

[8] Fanelli, G.C. & Larson, R.V. (2002) Practical management of posterolateral instability of the knee. Arthroscopy: The Journal of Arthroscopic & Related Surgery, 18, 1–8. Available from: 10.1053/jars.2002.31779 11828342

[9] Figueroa, F. , Figueroa, D. , Putnis, S. , Guiloff, R. , Caro, P. & Espregueira‐Mendes, J. (2021) Posterolateral corner knee injuries: a narrative review. EFORT Open Reviews, 6, 676–685. Available from: 10.1302/2058-5241.6.200096 34532075 PMC8419800

[10] Gwinner, C. , Weiler, A. , Roider, M. , Schaefer, F.M. & Jung, T.M. (2017) Tibial slope strongly influences knee stability after posterior cruciate ligament reconstruction: a prospective 5‐ to 15‐year follow‐up. The American Journal of Sports Medicine, 45, 355–361. Available from: 10.1177/0363546516666354 27651396

[11] Hammoud, S. , Reinhardt, K.R. & Marx, R.G. (2010) Outcomes of posterior cruciate ligament treatment: a review of the evidence. Sports Medicine and Arthroscopy Review, 18, 280–291. Available from: 10.1097/JSA.0b013e3181eaf8b4 21079509

[12] Harner, C.D. & Höher, J. (1998) Evaluation and treatment of posterior cruciate ligament injuries. The American Journal of Sports Medicine, 26, 471–482. Available from: 10.1177/03635465980260032301 9617416

[13] Herbst, E. , Muhmann, R.J. , Raschke, M.J. , Katthagen, J.C. , Oeckenpöhler, S. , Wermers, J. et al. (2023) The anterior fibers of the superficial MCL and the ACL restrain anteromedial rotatory instability. The American Journal of Sports Medicine, 51, 2928–2935. Available from: 10.1177/03635465231187043 37503921

[14] Jung, T.M. , Reinhardt, C. , Scheffler, S.U. & Weiler, A. (2006) Stress radiography to measure posterior cruciate ligament insufficiency: a comparison of five different techniques. Knee Surgery, Sports Traumatology, Arthroscopy, 14, 1116–1121. Available from: 10.1007/s00167-006-0137-3 16799824

[15] LaPrade, C.M. , Civitarese, D.M. , Rasmussen, M.T. & LaPrade, R.F. (2015) Emerging updates on the posterior cruciate ligament: a review of the current literature. The American Journal of Sports Medicine, 43, 3077–3092. Available from: 10.1177/0363546515572770 25776184

[16] LaPrade, R.F. , Wentorf, F.A. , Fritts, H. , Gundry, C. & Hightower, C.D. (2007) A Prospective magnetic resonance imaging study of the incidence of posterolateral and multiple ligament injuries in acute knee injuries presenting with a hemarthrosis. Arthroscopy: The Journal of Arthroscopic & Related Surgery, 23, 1341–1347. Available from: 10.1016/j.arthro.2007.07.024 18063179

[17] Li, L. , Li, J. , Zhou, P. , He, Y. , Li, Y. , Deng, X. et al. (2023) Decreased medial posterior tibial slope is associated with an increased risk of posterior cruciate ligament rupture. Knee Surgery, Sports Traumatology, Arthroscopy, 31, 2966–2973. Available from: 10.1007/s00167-023-07308-z 36622419

[18] Lundquist, R.B. , Matcuk Jr., G.R. , Schein, A.J. , Skalski, M.R. , White, E.A. , Forrester, D.M. et al. (2015) Posteromedial corner of the knee: the neglected corner. Radiographics, 35, 1123–1137. Available from: 10.1148/rg.2015140166 26172356

[19] Nedaie, S. , Vivekanantha, P. , O'Hara, K. , Slawaska‐Eng, D. , Cohen, D. , Abouali, J. et al. (2024) Decreased posterior tibial slope is a risk factor for primary posterior cruciate ligament rupture and posterior cruciate ligament reconstruction failure: a systematic review. Knee Surgery, Sports Traumatology, Arthroscopy, 32, 167–180. Available from: 10.1002/ksa.12025 38226729

[20] Ni, Q.K. , Song, G.Y. , Zhang, Z.J. , Zheng, T. , Cao, Y.W. & Zhang, H. (2022) Posterior tibial slope measurements based on the full‐length tibial anatomic axis are significantly increased compared to those based on the half‐length tibial anatomic axis. Knee Surgery, Sports Traumatology, Arthroscopy, 30, 1362–1368. Available from: 10.1007/s00167-021-06605-9 33977310

[21] Pacheco, R.J. , Ayre, C.A. & Bollen, S.R. (2011) Posterolateral corner injuries of the knee. The Journal of Bone & Joint Surgery British, 93–B, 194–197. 10.1302/0301-620X.93B2.25774 21282758

[22] Park, H.G. & Ham, H.J. (2020) Effect of posteromedial corner injury on stability and second‐look arthroscopic findings after posterior cruciate ligament reconstruction using allograft. Journal of Orthopaedics, 22, 104–108. Available from: 10.1016/j.jor.2020.03.042 32300271 PMC7153025

[23] Petersen, W. , Loerch, S. , Schanz, S. , Raschke, M. & Zantop, T. (2008) The role of the posterior oblique ligament in controlling posterior tibial translation in the posterior cruciate ligament‐deficient knee. The American Journal of Sports Medicine, 36, 495–501. Available from: 10.1177/0363546507310077 18182651

[24] Pizza, N. , Di Paolo, S. , Grassi, A. , Pagano, A. , Viotto, M. , Dal Fabbro, G. et al. (2023) Good long‐term patients reported outcomes, return‐to‐work and return‐to‐sport rate and survivorship after posterior cruciate ligament (PCL)‐based multiligament knee injuries (MLKI) with posteromedial corner tears as significant risk factor for failure. Knee Surgery, Sports Traumatology, Arthroscopy, 31, 5018–5024. Available from: 10.1007/s00167-023-07547-0 PMC10598146 37668614

[25] Rasenberg, E.I.J. , Lemmens, J.A.M. , van Kampen, A. , Schoots, F. , Bloo, H.J.K.C. , Wagemakers, H.P.A. et al. (1995) Grading medial collateral ligament injury: comparison of MR imaging and instrumented valgus‐varus laxity test‐device. A prospective double‐blind patient study. European Journal of Radiology, 21, 18–24. Available from: 10.1016/0720-048X(95)00660-I 8654454

[26] Ritchie, J.R. , Bergfeld, J.A. , Kambic, H. & Manning, T. (1998) Isolated sectioning of the medial and posteromedial capsular ligaments in the posterior cruciate ligament‐deficient knee. The American Journal of Sports Medicine, 26, 389–394. Available from: 10.1177/03635465980260030801 9617401

[27] Robinson, J.R. , Bull, A.M.J. , Thomas, R.R. & Amis, A.A. (2006) The role of the medial collateral ligament and posteromedial capsule in controlling knee laxity. The American Journal of Sports Medicine, 34, 1815–1823. Available from: 10.1177/0363546506289433 16816148

[28] Svantesson, E. , Hamrin Senorski, E. , Alentorn‐Geli, E. , Westin, O. , Sundemo, D. , Grassi, A. et al. (2019) Increased risk of ACL revision with non‐surgical treatment of a concomitant medial collateral ligament injury: a study on 19,457 patients from the Swedish National Knee Ligament Registry. Knee Surgery, Sports Traumatology, Arthroscopy, 27, 2450–2459. Available from: 10.1007/s00167-018-5237-3 PMC6656795 30374568

[29] Tibor, L.M. , Marchant Jr., M.H. , Taylor, D.C. , Hardaker Jr., W.T. , Garrett Jr., W.E. & Sekiya, J.K. (2011) Management of medial‐sided knee injuries, part 2: posteromedial corner. The American Journal of Sports Medicine, 39, 1332–1340. Available from: 10.1177/0363546510387765 21173192

[30] Toyooka, S. , Persson, A. , LaPrade, R.F. , Engebretsen, L. & Moatshe, G. (2023) Injury patterns in posterolateral corner knee injury. Orthopaedic Journal of Sports Medicine, 11, 23259671231184468. Available from: 10.1177/23259671231184468 37663094 PMC10469253

[31] Von Rehlingen‐Prinz, F. , Leiderer, M. , Dehoust, J. , Dust, T. , Kowald, B. , Frosch, K.H. et al. (2023) Association of medial collateral ligament complex injuries with anterior cruciate ligament ruptures based on posterolateral tibial plateau injuries. Sports Medicine – Open, 9, 70. Available from: 10.1186/s40798-023-00611-6 37553489 PMC10409938

[32] Weimann, A. , Schatka, I. , Herbort, M. , Achtnich, A. , Zantop, T. , Raschke, M. et al. (2012) Reconstruction of the posterior oblique ligament and the posterior cruciate ligament in knees with posteromedial instability. Arthroscopy: The Journal of Arthroscopic & Related Surgery, 28, 1283–1289. Available from: 10.1016/j.arthro.2012.02.003 22541643

[33] Willinger, L. , Balendra, G. , Pai, V. , Lee, J. , Mitchell, A. , Jones, M. et al. (2022) High incidence of superficial and deep medial collateral ligament injuries in ‘isolated’ anterior cruciate ligament ruptures: a long overlooked injury. Knee Surgery, Sports Traumatology, Arthroscopy, 30, 167–175. Available from: 10.1007/s00167-021-06514-x PMC8800884 33661325

[34] Willinger, L. , Shinohara, S. , Athwal, K.K. , Ball, S. , Williams, A. & Amis, A.A. (2020) Length‐change patterns of the medial collateral ligament and posterior oblique ligament in relation to their function and surgery. Knee Surgery, Sports Traumatology, Arthroscopy, 28, 3720–3732. Available from: 10.1007/s00167-020-06050-0 PMC7669796 32483671

[35] Yin, B. , Zhao, P. , Chen, J. , Yan, W. , Zhang, H. , Zhang, J. et al. (2022) Decreased lateral posterior tibial slope and medial tibial depth are underlying anatomic risk factors for posterior cruciate ligament injury: a case‐control study. BMC Musculoskeletal Disorders, 23, 689. Available from: 10.1186/s12891-022-05653-7 35858843 PMC9297602

[36] Yoo, J.H. , Chang, C.B. , Shin, K.S. , Seong, S.C. & Kim, T.K. (2008) Anatomical references to assess the posterior tibial slope in total knee arthroplasty: a comparison of 5 anatomical axes. The Journal of Arthroplasty, 23, 586–592. Available from: 10.1016/j.arth.2007.05.006 18514879

[37] Yoon, K.H. , Lee, J.‐H. , Kim, S.‐G. , Park, J.‐Y. , Lee, H.‐S. , Kim, S.J. et al. (2023) Effect of posterior tibial slopes on graft survival rates at 10 years after primary single‐bundle posterior cruciate ligament reconstruction. The American Journal of Sports Medicine, 51, 1194–1201. Available from: 10.1177/03635465231156621 36927119

